# Simulation Improves Internal Medicine Resident Confidence With Defibrillation, Cardioversion, and Transcutaneous Pacemaker Use

**DOI:** 10.7759/cureus.16648

**Published:** 2021-07-26

**Authors:** Alexander W Smith, John O Elliott, Brad D Gable

**Affiliations:** 1 Internal Medicine, Riverside Methodist Hospital, Columbus, USA; 2 Medical Education, OhioHealth Research Institute, Columbus, USA; 3 Medical Simulation, OhioHealth, Columbus, USA

**Keywords:** simulation based medical education, graduate medical education, clinical skills practice, advanced cardiac life support, critical care

## Abstract

Introduction

While many graduate medical education programs require residents to be certified in advanced cardiac life support, this does not cover all aspects of cardiac stabilization in patients with a pulse. Residents are often on the front lines of providing care to patients with life-threatening dysrhythmias. Our residents expressed a lack of confidence in their ability to provide this care.

Methods

A convenience sample of internal medicine, preliminary medicine, and transitional year residents from our large community-based tertiary care hospital participated in our survey and training. We utilized a pre-post survey method of our residents’ confidence in domains that are critical to caring for patients requiring cardiac resuscitation and stabilization. Our pre-post survey was a modified Likert scale. Our training consisted of a 1-hour faculty-led hands-on training session focused on these critical domains in our hospital’s simulation suites. Follow-up survey data were collected immediately after the training and at six and 11 months after the training using mean confidence across all five domains as the study variable.

Results

Resident mean confidence in the five domains (placing leads and pads, manipulating defibrillator controls, performing defibrillation, performing synchronized cardioversion, and performing transcutaneous pacemaker use) increased immediately after our training compared to before the training (p<0.001). This increase in confidence from before the training was sustained at six and 11 months after the training (p=0.001 and p=0.002, respectively). Confidence was lower at six and 11 months than immediately after training (p=0.01 and p=0.004, respectively).

Conclusion

Our project showed that simulation-based training was effective in improving our trainee’s confidence in providing care to patients with life-threatening dysrhythmias. As with previous studies in simulation, confidence degradation was seen over time and likely mirrors skill degradation in these low-frequency encounters. As such, future aims include identification of ideal time intervals between training.

## Introduction

Many residents are required to maintain certification in advanced cardiac life support (ACLS). Often, residents are the primary providers in emergency departments, intensive care units, clinics, and hospitals at training institutions around the country. Each institution has unique characteristics that make education for cardiac emergencies challenging. These include patient population, type/models of defibrillators, and nursing/associate ACLS and basic life support (BLS) training and certification. Thus, prior training and education with one make or model or at a different institution may not always translate between training sites and places of employment. Despite this, residents remain on the front lines of managing patients critically ill with unstable and life-threatening tachydysrhythmias and bradydysrhythmias.

Increasing concern has been raised in the medical literature regarding decreasing exposure to cardiac arrest scenarios in the hospital [1]. These concerns have been met with calls for increased educational structure and high-quality simulation training [2]. These same concerns extend to life-threatening tachydysrhythmias and bradydysrhythmias that are commonly tested but rarely encountered by physicians in training.

Simulation training has been shown to be effective in raising resident confidence levels in being effective leaders of ACLS [3]. Our internal medicine residency program provides regular simulation training for ACLS scenarios, but identified that our residents lacked comfort with providing care to patients with life-threatening dysrhythmias with a pulse. Many also reported a lack of familiarity with the defibrillator equipment for uses other than defibrillation during cardiac arrest. We sought to evaluate the effects that a one-time simulation-based education would have on resident confidence, and the retention of that confidence over time, in providing this advanced care to critically ill patients.

## Materials and methods

A convenience sample of internal medicine, preliminary medicine, and transitional year residents from our large community-based tertiary care hospital participated in our survey and training. We utilized a pre-post survey method of our residents’ confidence in domains that are critical to caring for patients requiring cardiac resuscitation and stabilization. Our electronically distributed pre-post survey was collected via the REDCap survey application service and utilized a modified Likert scale evaluating resident confidence in five domains deemed critical to providing care to patients acutely and critically ill with life-threatening dysrhythmias at risk of cardiac death without emergent intervention. These domains were determined as the educational objective for our education by our internal medicine and critical care faculty and content experts. Residents completed a Likert scale of strongly disagree, disagree, neutral, agree, or strongly agree each of which was assigned a numerical value of 1-5, respectively. Resident-self-reported confidence was obtained regarding their ability to perform each of the five critical functions: properly place defibrillator leads and pads on a patient, manipulate the defibrillator controls to attain desired settings, perform defibrillation, perform synchronized cardioversion, and perform transcutaneous pacing.

Following this survey, an hour-long hands-on training session was provided to all available residents in our hospital simulation suites. During this education, faculty instructors guided residents through several cases where the residents were provided feedback on performing the aforementioned five functions. Additionally, residents were able to manipulate settings on the defibrillators and evaluate the resultant effects on their simulated patients.

Residents were then surveyed immediately after the training session to re-evaluate their confidence in the five critical functions previously stated. Residents were again surveyed at six and 11 months after the training session to evaluate whether any changes in confidence were lasting and evaluate if the education was being applied in the clinical setting. These time intervals were determined using our standardized data-collection plan, and Return on Investment in Learning protocols [4].

Each resident’s confidence in the five domains was scaled numerically from 1 (strongly disagree) to 5 (strongly agree). Their confidence in the five domains was then added together on a scale of 5 to 25. The individual summated confidences were then averaged for all of the residents who were surveyed before, immediately after and six months after the training session. These mean total confidence scores were compared between each of the three survey groups.

## Results

**Figure 1:**
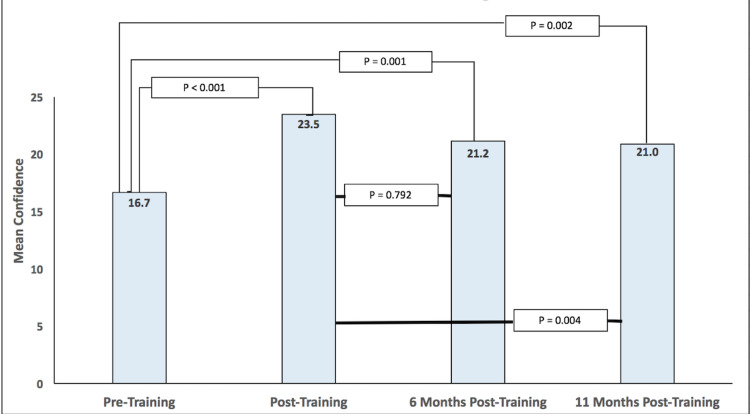
Pacing/Defibrillation/Cardioversion Training

Among the 38 residents who were available for the training session and completed the initial confidence survey, the mean confidence rating in the five domains was 16.7 (95% CI: 14.9-18.5). As seen in Figure 1, immediately after the training session, this mean confidence rating in the five domains among 33 survey respondents who participated in the session and responded to the follow-up survey was 23.5 (95% CI: 22.4-24.6). Five participants did not complete the initial follow-up survey. There was statistical significance in this difference with a p-value of <0.001. Six months after the training session, the mean confidence rating in the five domains was 21.2 (95% CI: 19.8-22.6) among 20 participating residents, statistically different than the pre-training confidence (p=0.001). At 11 months out from the training, the mean confidence among 21 participants was 21.0 (95% CI: 19.6-22.4), again significantly higher than the confidence prior to the training (p=0.002). At both six and 11 months out from the training the mean confidence rating was significantly lower than immediately after training (p=0.01 and p=0.004, respectively). There was an additional loss of survey respondents noted at the six- and 11-month follow-up period.

Several residents noted that they had applied skills learned in our session during actual patient care on the follow-up surveys, while the others stated they had not had an opportunity to apply the skills in the clinical setting.

## Discussion

Simulation has become a mainstay of medical education, allowing learners to experience high-risk low-frequency situations in a safe learning environment [5]. Cardiopulmonary resuscitation (CPR), specifically, has been identified as one of the biggest opportunities for simulation-based education in graduate medical education [6]. Our project showed that simulation-based education following a deliberate practice model for residents improved their confidence in providing defibrillation, synchronized cardioversion, and transcutaneous pacemaker utilization to critically ill patients they may encounter in our large tertiary care center. Deliberate practice is a cornerstone in medical simulation education. Through deliberate practice learners are paired with master educators and are given immediate feedback to continually refine their skills until mastery is achieved [7-10]. To that end, educators in this simulation followed a deliberate practice model of education. This led to participants being able to perform each procedure multiple times until proficiency was achieved. In addition, learners were able to receive clinical feedback from the mannequin and monitors as different interventions were performed. This included rhythm changes, pulse changes, blood pressure changes, and electric and mechanical capture with transcutaneous pacing. 

An ongoing question in medical education related to simulation is that of skill degradation and the role simulation may play in abating said degradation. Studies have demonstrated wide variation of results when examining skill degradation. Specific to resuscitation, some studies of BLS and ACLS skills have noted relatively short-term degradation of skills (from three months to 12 months) [11-16]. Other studies meanwhile have suggested that degradation may not occur until 18 months [17] and that skill retention up to 80% may still be present at 12 months [18]. A systematic review and meta-analysis looking at the topic found a paucity of high-quality randomized controlled trials confirming these otherwise conflicting conclusions, confounded by significant heterogeneity and lack of methodological consistency [19]. Accepting the variations of skill degradation noted in prior studies, the confidence degradation seen in our study generally correlates with the previously demonstrated skill degradation for BLS and ACLS skills. The major limitation of our study is the primary outcome being confidence scores rather than objective measures of skill retention but given the degradation of confidence seen we feel strongly that this coincides with skill degradation over the same time course.

The optimum frequency of education for CPR and use of automated electronic defibrillators is yet to be identified [20,21]. Again this is likely based on a relative paucity and inconsistency of evidence surrounding optimal timing of refresher courses to prevent skills degradation. In relation to the skills we evaluated, one notable study did demonstrate improved time to onset of chest compressions and time to defibrillation for in-hospital cardiac arrest for non-ICU nurses with short refresher simulation trainings at two and three months as compared to six months for a control group without refresher training [22]. Further study is needed in this area and attention to multiple groups is needed; from nurses providing initial ACLS care, to providers who may be required to perform the higher-order skills discussed in our study.

Our findings add to the body of literature surrounding the value of simulation training as a viable and effective method of educating learners on the critical aspects of a skill and improving their confidence in performing that skill. In particular, we feel that simulation-based education is especially effective for training for low-frequency high-risk situations and procedures. As with previous work in the field of simulation training, our results did demonstrate improved confidence along with confidence degradation over time, which we expect likely mirrors skill degradation over that same time period. Our own future aims are to understand the optimal interval for re-education of our learners to prevent knowledge and skill degradation, with enhanced evaluations of quality of care delivery measures on re-assessment.

## Conclusions

Our study demonstrated clear improvements in confidence in the skills of defibrillation, cardioversion, and transcutaneous pacemaking with simulation-based training following a deliberate practice model led by master educators in our institution. Confidence degradation mirroring previously described skill degradation for similar skill training was observed. 

Simulation-based education is an effective method for improving resident confidence in utilizing low-frequency high-acuity skills like defibrillation, cardioversion, and transcutaneous pacemaking. Further studies are required to elucidate the timing of skill degradation in this arena to help guide training programs and other institutions with regard to provider refresher courses.
